# Do your patients with bipolar disorder use dietary supplements?

**DOI:** 10.1186/s40345-015-0031-3

**Published:** 2015-09-16

**Authors:** Peter C Whybrow, Tasha Glenn

**Affiliations:** Department of Psychiatry and Biobehavioral Sciences, Semel Institute for Neuroscience and Human Behavior, University of California Los Angeles (UCLA), 300 UCLA Medical Plaza, Los Angeles, CA 90095 USA; ChronoRecord Association Inc., PO Box 3501, Fullerton, CA 92834 USA

It is increasingly evident that physicians should routinely ask patients with bipolar disorder about the use of dietary supplements. In our recent study of 348 patients with bipolar disorder, about 3 out of 10 patients used dietary supplements in addition to prescribed drugs, with 2 in 10 of these taking supplements for the long term (Bauer et al. 2015). The patients tried about 40 different supplements, with fish oil, vitamin B preparations, multivitamins, and melatonin taken most frequently. Dietary supplements have the potential to interfere with the treatment of bipolar disorder due to patient misconceptions, and the safety and quality of some products.

Patients may turn to dietary supplements to counteract side effects of prescribed drugs or because of a poor outcome. The majority of dietary supplements are self-selected, rather than recommended by a physician, and product choice is primarily influenced by friends, testimonials, aggressive marketing claims, price, and traditions (OIG 2012; New York State 2005). Many patients have misconceptions about dietary supplements, such as that “natural” is synonymous for safe, that supplements are safer than prescribed drugs, that megadoses are safe, and that the products and advertisements are pre-approved by the FDA.

Dietary supplements may interact with prescription drugs or with other dietary supplements (Izzo and Ernst 2009). Most interactions with dietary supplements reported in the literature involve prescription drugs that affect the central nervous system or cardiovascular system (Tsai et al. 2012). Patients with bipolar disorder may also be at increased risk of interactions because they often take multiple prescription drugs. Another concern is that some dietary supplements may potentially cause psychiatric adverse reactions such as anxiety, panic attack, confusion, hallucinations, or mania (Ernst 2003). The FDA estimates that there is extensive underreporting of adverse events from dietary supplements (GAO 2013).

Many quality problems have been reported for dietary supplements, including product contamination, ingredients not matching those listed on the label, and dosage strength inconsistencies. Between 2004 and 2012, of all FDA drug recalls due to risk of serious adverse consequences or death, over half were for dietary supplements (Harel et al. 2013). Analytical testing of the ingredients in many dietary supplements manufactured in the US found the strength varied widely from that listed on the label (Lockwood 2011). In 2015, an investigation by the New York State Attorney General’s office found that 80 % of store brand herbal dietary supplements at 4 large national chain stores did not contain the herbs listed on the label (O’Connor 2015). In addition, enforcement of current good manufacturing practices by the FDA is limited, with only 11 % of the dietary supplement manufacturing facilities inspected in fiscal year 2013 (Long 2014).

The evidence suggests that physicians should assume that patients will not volunteer information on the use of supplements and that this may not be included in a patient's electronic medical record. To provide optimal treatment of bipolar disorder, physicians need to understand patient use of dietary supplements.
